# Regulation of endothelial ferroptosis by SESN1 in atherosclerosis and its related mechanism

**DOI:** 10.18632/aging.204777

**Published:** 2023-06-08

**Authors:** Feng Gao, Bin Zhang, Zhanfa Sun, Yuan Gao, Chunyi Liu, Xueyong Dou, Haokun Tong, Rui Wang

**Affiliations:** 1 Department of Cardiovascular Surgery, Xuzhou Cancer Hospital, Xuzhou, Jiangsu 221005, China

**Keywords:** SESN1, AS, ferroptosis, P21, inflammation

## Abstract

Background: Atherosclerosis (AS) is a disease characterized by the disorder of lipid metabolism and the formation of atherosclerotic plaques in the arterial wall, leading to arterial stenosis. Sestrins 1 (SESN1) plays an important regulatory role in AS, but the specific regulatory mechanism is still unclear.

Methods: ApoE−/− mouse models of AS were constructed. After overexpressing SESN1, oil red O staining was used to detect the degree of aortic plaque. HE staining detected the endothelial damage of the surrounding tissues. ELISA was used to detect the levels of vascular inflammation and oxidative stress. The iron metabolism in vascular tissues was detected by immunofluorescence. The expressions of SESN1 and ferroptosis-related proteins were detected by western blot. In the oxidized low-density lipoprotein (ox-LDL)-induced injury model in human umbilical vein endothelial cells (HUVECs), CCK8, ELISA, immunofluorescence and western blot were respectively used to detect cell viability, inflammatory response, oxidative stress and ferroptosis. The regulatory mechanism of SESN1 on endothelial ferroptosis in AS was further explored following the addition of P21 inhibitor UC2288.

Results: Overexpression of SESN1 could inhibit the extent of the plaque and reduce the endothelial injury of plaque tissues in AS mice. In both mouse and cell models of AS, SESN1 overexpression inhibited inflammatory response, oxidative stress response, and endothelial ferroptosis. The inhibitory effect of SESN1 on endothelial ferroptosis might be achieved through activation of P21.

Conclusion: SESN1 overexpression plays an inhibitory role in vascular endothelial ferroptosis through the activation of P21 in AS.

## INTRODUCTION

Atherosclerosis (AS) is a disease characterized by arterial stenosis on account of lipid metabolism disorder and AS plaque formation in the arterial wall [1, 2]. In the process of AS, thrombosis is superimposed on ruptured or eroded atherosclerotic plaques, resulting in blood flow restriction or blockade, thereby accelerating the occurrence and development of life-threatening vascular diseases such as stroke [3]. Among many cardiovascular diseases, ischemic heart disease due to AS plaque rupture accounts for 42.5% of mortality, and the rate of cardiovascular-related deaths is increasing [4]. Therefore, it is particularly important to explore the pathogenesis of AS.

Ferroptosis is a novel mode of cell death characterized by iron-dependent lipid peroxidation accompanied by decreased glutathione peroxidase 4 (GPX4) activity, ultimately leading to oxidative stress and cell death [5]. The previous study has shown that in the process of lipid peroxidation metabolism, large amounts of ROS and lipid peroxides in the body aggravate oxidative stress, cause endothelial dysfunction and activate inflammatory reaction, which not only stimulates ferroptosis but also induces the occurrence and development of vascular diseases such as AS [6]. In ApoE−/− mice, iron overload leads to endothelial dysfunction by enhancing oxidative stress and inflammatory responses, thereby accelerating the progression of AS [7–9]. Therefore, the regulation of endothelial ferroptosis may be a breakthrough point in the treatment of AS.

Sestrins (SESN) are a family of highly conserved stress response proteins, including three family members SESN1, SESN2 and SESN3. Among them, SESN1 is transcriptionally regulated by P53 and forkhead transcription factors, exhibits oxidoreductase activity *in vivo*, and protects cells from oxidative stress [10, 11]. Rapamycin attenuates stress-induced endothelial apoptosis through mTOR and SESN1-related redox regulation [12]. SESN1 is highly expressed in aortic macrophages of mice in the AS model and inhibits LPS-induced NLRP3 inflammasome activation and NK-κB signaling pathway activation in macrophages [13]. Our previous study also showed that activation of SESN1 attenuated ox-LDL-induced macrophage inflammation and lipid accumulation [14]. However, few studies are available regarding the role of SESN1 in ferroptosis. Importantly, the study has reported that SESN2 plays a tumor-promoting role in iron-rich environments by inhibiting oxidative stress-related cell death in colon cancer [15]. Moreover, SESN1 and SESN2 share nearly 50% of the same amino acid sequence, showing high similarity (33). Therefore, we hypothesize that SESN1 has a regulatory role in vascular endothelial ferroptosis in AS.

## MATERIALS AND METHODS

### Database

P21 was predicted to be a downstream target gene of SESN1 by the STRING website (https://cn.string-db.org/).

### AS mouse model

Male C57BL/6J mice and ApoE−/− mice (6–8 weeks old, 20–22 g) were housed in a temperature-controlled environment (21 ± 1°C) with (40–60%) humidity with a 12 hours light/dark cycle, and were provided free access to tap water and regular chow. All experimental protocols were approved by the Animal Care and Use Committee of Xuzhou Cancer Hospital. The mice were randomly assigned to control group (*n* = 5 C57BL/6J mice) and AS group (model, *n* = 5 ApoE−/− mice). Mice model of AS was established by feeding mice a high-fat diet plus VitD3 (3 × 10^5^ U/kg) for 8 weeks as previously described [16]. At the same time, SESN1 overexpression (Ov-SESN1) plasmid (1.0 × 10^9^ TU/ml, 10 μl, from Rebo Biota) was injected into ApoE−/− mice via tail vein (*n* = 5). ApoE−/− mice in Ov-NC group received the same dose of normal saline intravenously (*n* = 5). After 8 weeks, blood was collected from the orbit and tissue was removed for subsequent analysis [17].

### Quantitative reverse transcription-polymerase chain reaction (qRT-PCR)

Total RNA from mice aortic tissues was extracted via TRIzol reagent (Invitrogen, Carlsbad, CA, USA), according to the instructions. PrimeScript RT Master Mix (TaKaRa, Shiga, Japan) was used to prepare cDNA and qRT-PCR was performed on a Light Cycler^®^ 480 with the SYBR Green I Master Kit (Roche, Basel, Switzerland) to detect mRNA expression. The relative expression levels were calculated through the 2^−ΔΔCt^ method.

### Oil red O staining

Mice aortic tissues were fixed in 10% phosphate-buffered formalin for 10 min, and then rinsed in 60% isopropanol for 15 sec. Cells were stained with Oil Red O solution (Sigma-Aldrich) at 37°C for 10 min in darkness, followed by a rinse with 60% isopropanol for 15 sec. Cells were then incubated in hematoxylin solution (Sigma-Aldrich) for 1 min, rinsed with PBS, and observed under a Zeiss light microscope.

### Hematoxylin and eosin (HE) staining

For histopathological analysis, the surrounding plaque tissues were fixed in 4% paraformaldehyde (PFA) and embedded in paraffin. 5-micron sections were placed onto glass slides and stained with HE. Finally, the endothelial injury of the surrounding tissues of the plaque was observed.

### ELISAs

The levels of IL-6, IL-1β and TNF-α in vascular tissues were detected via the corresponding ELISA kits (Nanjing Jiancheng, Nanjing, China) according to the instructions. The absorbance was measured at 450 nm using a microplate reader (Varioskan Flash, Thermo Fisher Scientific, USA).

### Detection of reactive oxygen species (ROS), malonaldehyde (MDA) and glutathione (GSH)

The ROS level was determined by 2′,7′-dichlorofluorescein diacetate (DCFH-DA) assay. Briefly, aortic tissue homogenate in PBS was incubated with 10 μM DCFH-DA probe. Fluorescence intensity was measured by a SpectraMax Gemini-EM microplate reader with a wavelength of 455 nm after a 30 min incubation at 37°C in dark. The MDA level was measured through detecting the red MDA-TBA (thiobarbituric acid) adduct. GSH was quantified by the formation of a yellow-colored product (5-thio-2-nitrobenzoic acid) generated from the reaction of the sulfhydryl group of GSH with DTNB (5,5′-dithio-bis-2-nitrobenzoic acid).

### Fe^2+^ and Fe^3+^ probe incubation and imaging

The vascular tissues were frozen to prepare the cryosections. A set of 20-μm-thick sections was obtained from the selected vascular region. Then the sections were incubated with 0.3% Triton X-100. And the 20 μM Fe^2+^ and Fe^3+^ probe 1 was added onto the sections, respectively. After 20 min incubation, each section was photographed under the confocal laser scanning microscope to visualize the probe 1-labeled Fe^2+^ and Fe^3+^.

### Western blot

Protein was extracted from tissues and cells in RIPA buffer for 30 min on ice. Protein concentrations were detected using the Pierce BCA Protein Assay kit (Thermo Fisher Scientific). Next, 30 mg protein were separated by electrophoresis on a sodium dodecyl sulfate-polyacrylamide gel, transferred to a PVDF membrane which was then blotted with 5% milk. The membranes were incubated with primary antibodies (1:1000, Abcam) for 2 h at 4°C and subsequently incubated with secondary antibodies (1:5000, Abcam) conjugated with peroxidase. The signal was then detected using Chemiluminescent Detection System.

### Cell culture

The human umbilical vein endothelial cells (HUVECs) obtained from BeNa Culture Collection (Henan, China) were cultured in DMEM medium (Gibco, Thermo Fisher Scientific) containing 10% fetal Bovine serum (FBS, Gibco). HUVECs were challenged with ox-LDL (100 μg/mL) for 24 h.

### Cell transfection

Overexpression of SESN1 (Ov-SESN1) and overexpression control (Ov-NC) were all synthesized by RIBOBIO (Guangzhou, China). Plasmid transfection was performed using Lipofectamine™ 2000 according to the manufacturer’s instructions. Efficiency of transfection was detected with RT-qPCR and western blot 24 h after transfection.

### Cell counting Kit-8 proliferation assay (CCK-8)

The cell viability of HUVECs was detected by CCK-8 kit according to the instructions. Briefly, HUVECs were seeded on 96-well plates and treated with ox-LDL. Then, 10 μL CCK-8 was added to each well for 2 h incubation at 37°C and absorbance was measured at 450 nm by Biotek microplate reader (Winooski, VT, USA).

### Statistical analysis

All data were analyzed with SPSS 26.0 software and expressed as mean ± standard deviation. Normal distribution data were compared using Student’s *t*-test (comparison between two groups) or analysis of variance (comparison between multiple groups). *P* < 0.05 indicates that the difference is statistically significant.

### Availability of data and materials

The analyzed data sets generated during the present study are available from the corresponding author on reasonable request.

## RESULTS

### Overexpression of SESN1 inhibited plaque formation in AS mice

**Figure 1:**
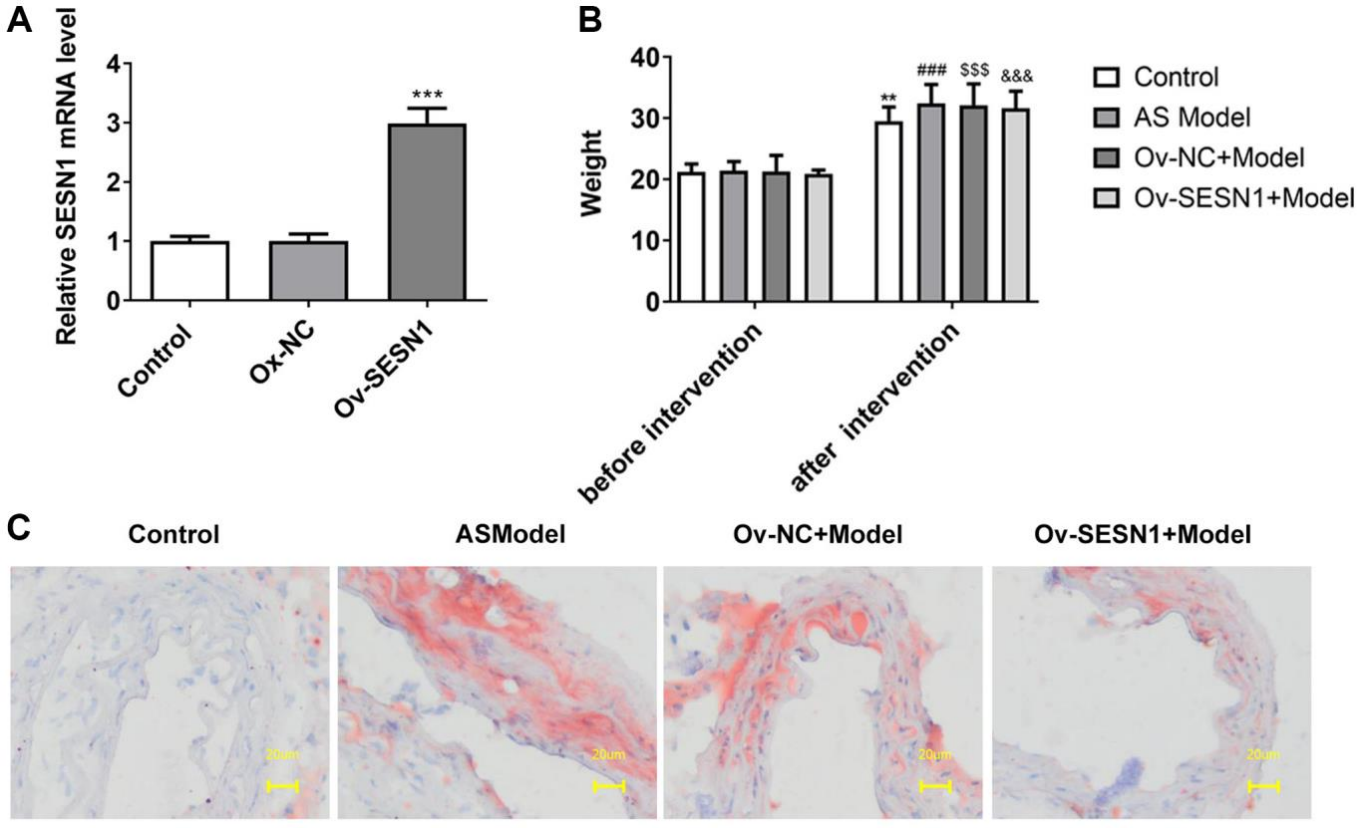
**Overexpression of SESN1 inhibited plaque formation in AS mice.** (**A**) RT-qPCR assayed the efficiency of SESN1 overexpression. ^***^P < 0.001 vs. Ox-NC. (**B**) The body weight of mice. (**C**) Oil Red O staining was used to detect the degree of aortic plaque. ^**^P < 0.01, ^###^*P* < 0.001, ^$$$^*P* < 0.001, ^&&&^*P* < 0.001 vs. before intervention.

RT-qPCR analyzed the transfection efficiency of SESN1 overexpression plasmids and it was showed that SESN1 expression was significantly increased after transfection of Ov-NC (Figure 1A). Mice were divided into control, AS model, Ov-NC+Model and Ov-SESN1+Model groups. After 8 weeks, the body weight of mice was increased, but the body weight of mice in the control group and Ov-SESN1+Model group was decreased slightly compared with the AS model group (Figure 1B). Oil Red O staining was used to detect the degree of aortic plaque, and the results showed that there were more aortic plaques in the AS model group compared with the control group. Plaque was reduced in the Ov-SESN1+Model group compared with the Ov-NC+Model group (Figure 1C).

### Overexpression of SESN1 inhibited inflammation and oxidative stress in AS mice

**Figure 2:**
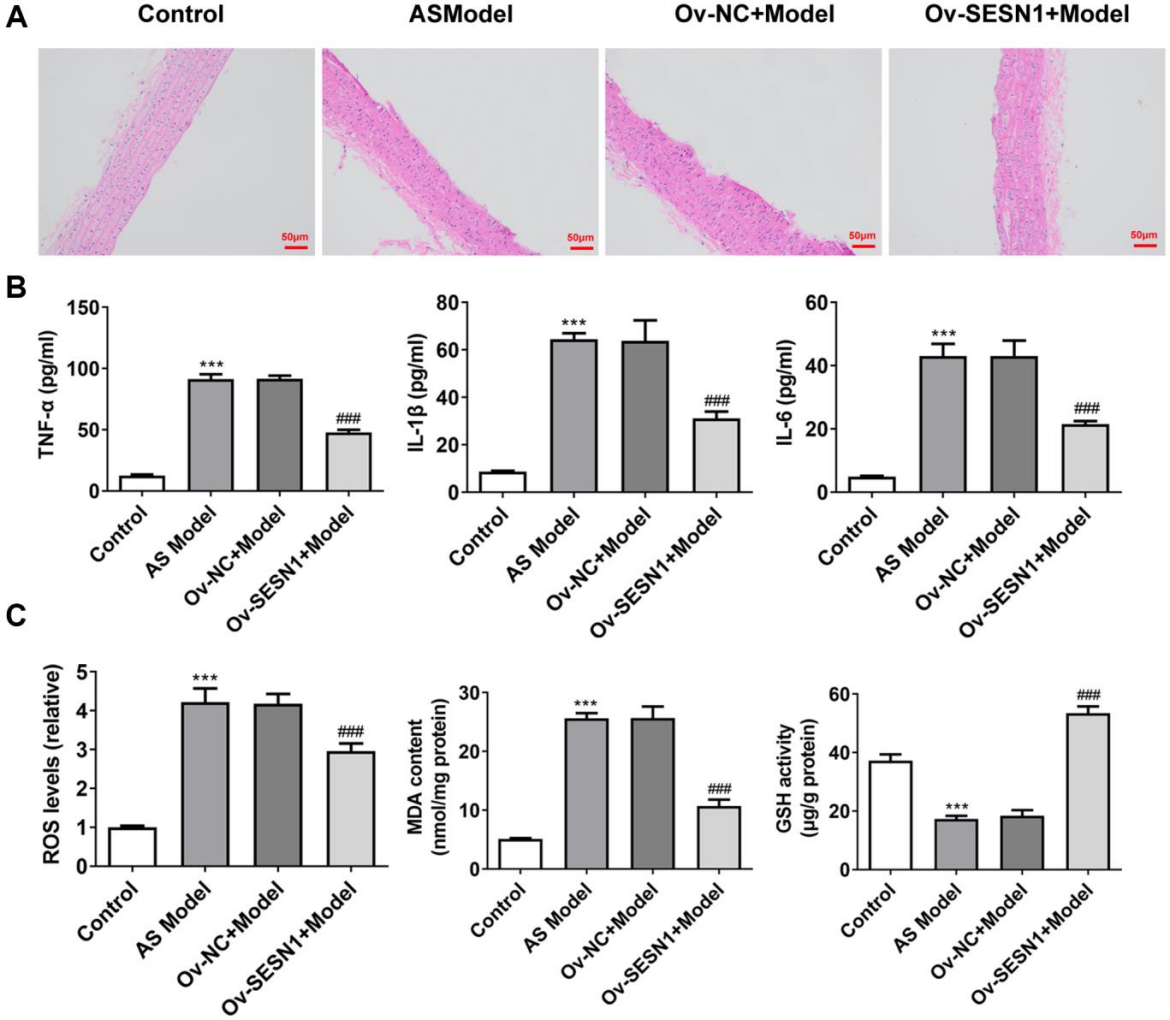
**Overexpression of SESN1 inhibited inflammation and oxidative stress in AS mice.** (**A**) The results of HE staining. (**B**) ELISA was used to detect the levels of inflammatory factors in vascular tissues. (**C**) The kits were used to detect the expression of oxidative stress-related factors ROS, MDA and GSH. ^***^P < 0.001 vs. Control; ^###^*p* < 0.001 vs. Ov-NC+Model.

The results of HE staining showed that there was no damage to the endothelium surrounding the plaques in the control group. Serious endothelial damage surrounding the plaques was noticed in the AS model group. Compared with Ov-NC+Model group, reduced endothelial damage in periplaque regions was observed in Ov-SESN1+Model group (Figure 2A). ELISA was used to detect the levels of inflammatory factors in vascular tissues, and the results showed that the expressions of IL-6, IL-1β and TNF-α in AS model group were significantly increased compared with those in control group. The levels of inflammatory cytokines were significantly inhibited in the Ov-SESN1+Model group compared with the Ov-NC+Model group (Figure 2B). Next, we used the kits to detect the expression of oxidative stress-related factors ROS, MDA and GSH, and the results showed that the expression of ROS and MDA in AS model was abnormally increased while the expression of GSH was abnormally decreased. Overexpression of SESN1 in the Model group reversed the expression of ROS, MDA and GSH (Figure 2C).

### Overexpression of SESN1 inhibited ferroptosis in AS mice

**Figure 3:**
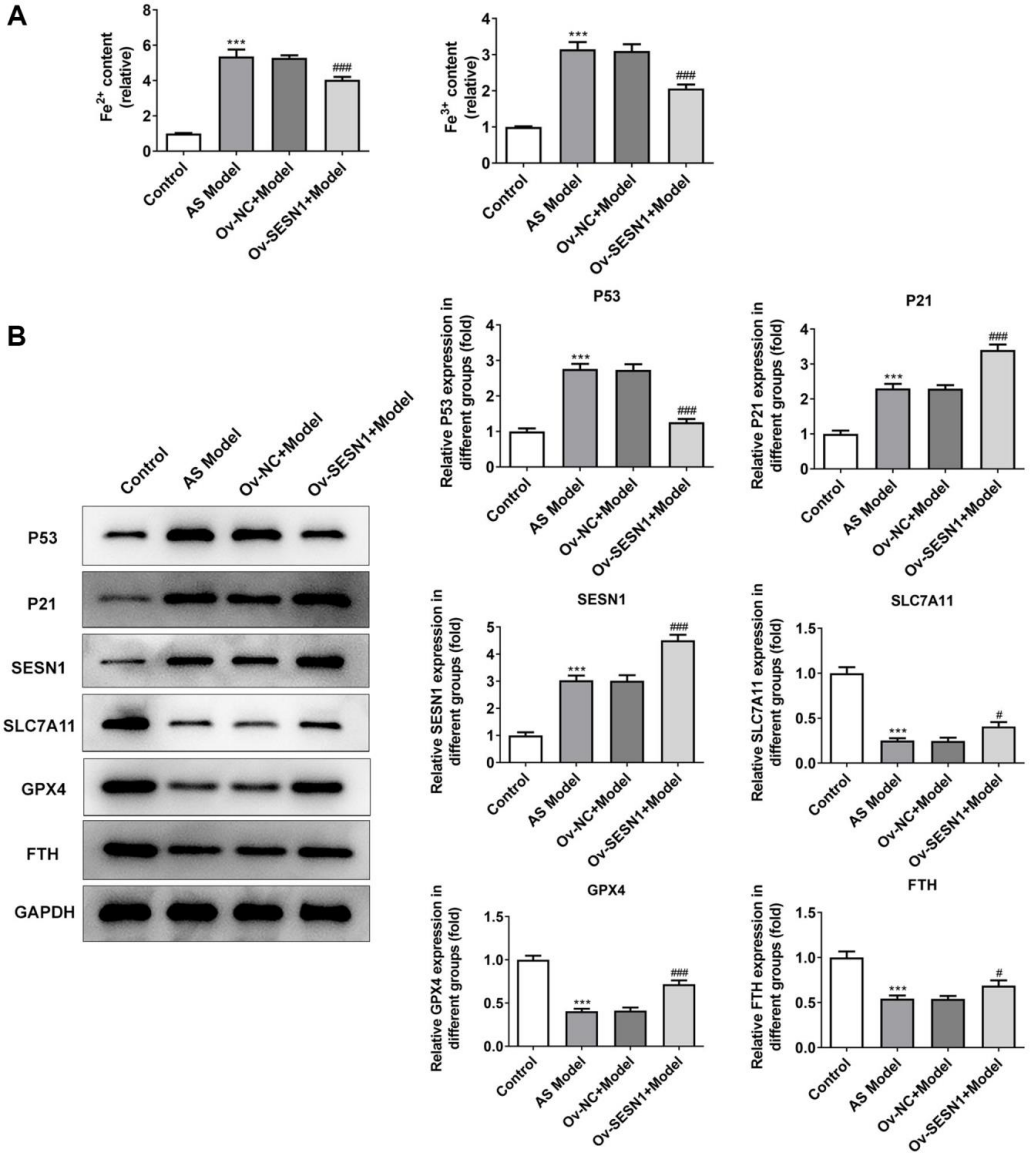
**Overexpression of SESN1 inhibited ferroptosis in AS mice.** (**A**) Immunofluorescence was used to detect the iron metabolism in vascular tissues. (**B**) The expressions of SESN1, P53, P21 and ferroptosis-related proteins were detected by western blot. ^***^P < 0.001 vs. Control; ^#^*P* < 0.05, ^###^*p* < 0.001 vs. Ov-NC+Model.

Immunofluorescence was used to detect the iron metabolism in vascular tissues. The results showed that the expression of Fe^2+^ and Fe^3+^ in vascular tissues of AS model group was significantly increased compared with that of control group. Overexpression of SESN1 significantly decreased Fe^2+^ and Fe^3+^ expression in the model group (Figure 3A). The expressions of SESN1 and ferroptosis-related proteins were detected by western blot. The results showed that compared with the control group, the expressions of SESN1, P53 and downstream P21 in the AS model group were significantly increased, and the expressions of ferroptosis-related proteins SLC7A11, GPX4 and FTH were significantly decreased. Compared with the Ov-NC+Model group, the expression of P53 was decreased, the expressions of P21 and SESN1 were significantly increased, and the expressions of ferroptosis-related proteins were significantly increased in the Ov-SESN1+Model group (Figure 3B).

### Overexpression of SESN1 increased cell activity and inhibited cellular inflammation and oxidative stress responses in ox-LDL-induced HUVECs

**Figure 4:**
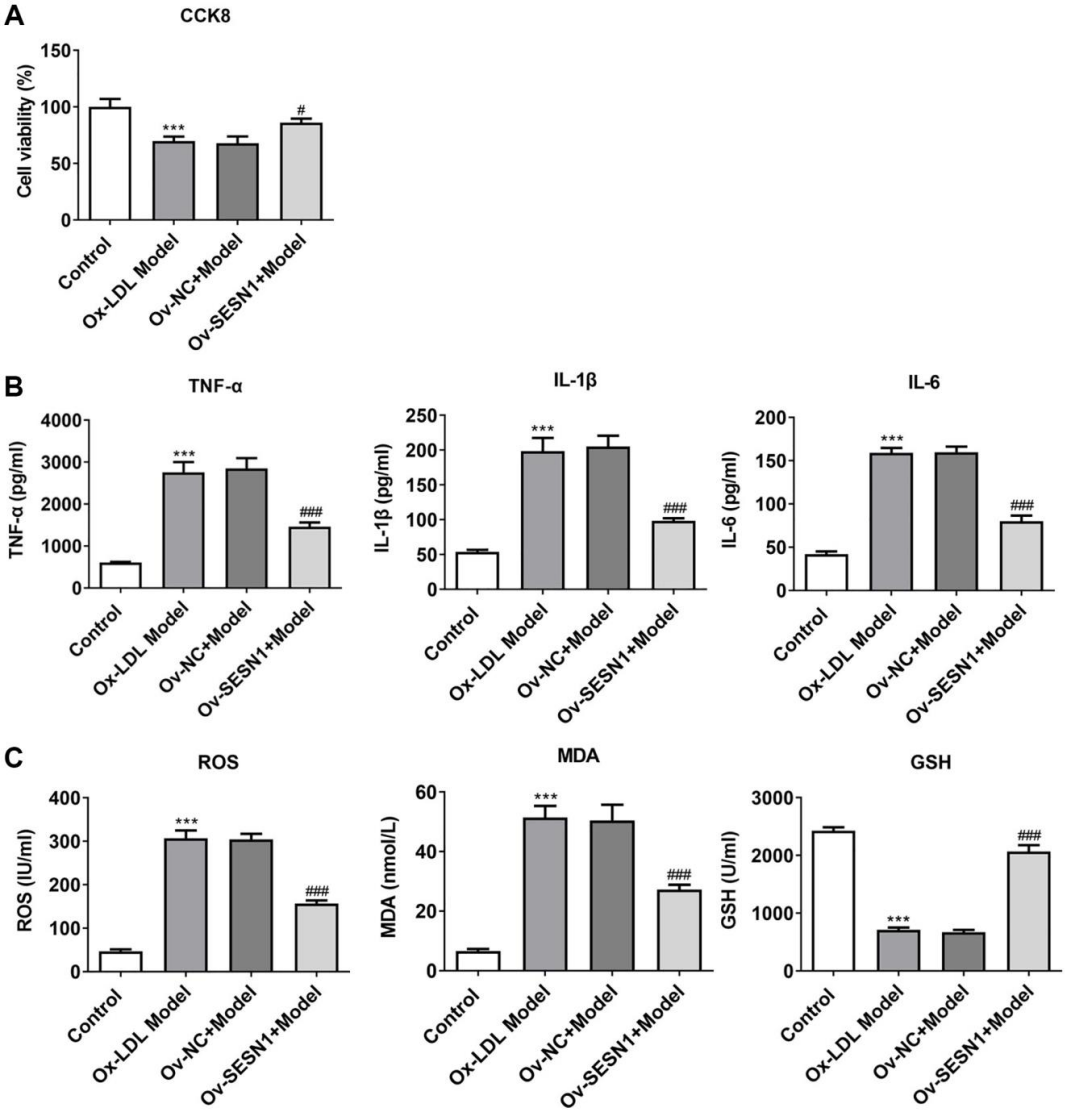
**Overexpression of SESN1 increased cell activity and inhibited cellular inflammation and oxidative stress responses in ox-LDL-induced HUVECs.** (**A**) CCK8 was used to detect the cell activity. (**B**) ELISA was used to detect the levels of inflammatory factors in cells. (**C**) The kits were used to detect the expression of oxidative stress-related factors ROS, MDA and GSH. ^***^P < 0.001 vs. Control; ^#^*P* < 0.05, ^###^*p* < 0.001 vs. Ov-NC+Model.

CCK8 results revealed that HUVECs viability was decreased significantly after exposure to ox-LDL, and increased significantly after further overexpression of SESN1 (Figure 4A). ELISA results showed that the expressions of IL-6, IL-1β and TNF-α in HUVECs induced by ox-LDL were significantly increased, while the expressions of inflammatory factors were inhibited after further overexpression of SESN1 (Figure 4B). The trend of ROS and MDA was consistent with the trend of the expression inflammatory factors, while the trend of GSH was opposite (Figure 4C).

### Overexpression of SESN1 inhibited ferroptosis in ox-LDL-induced HUVECs

**Figure 5:**
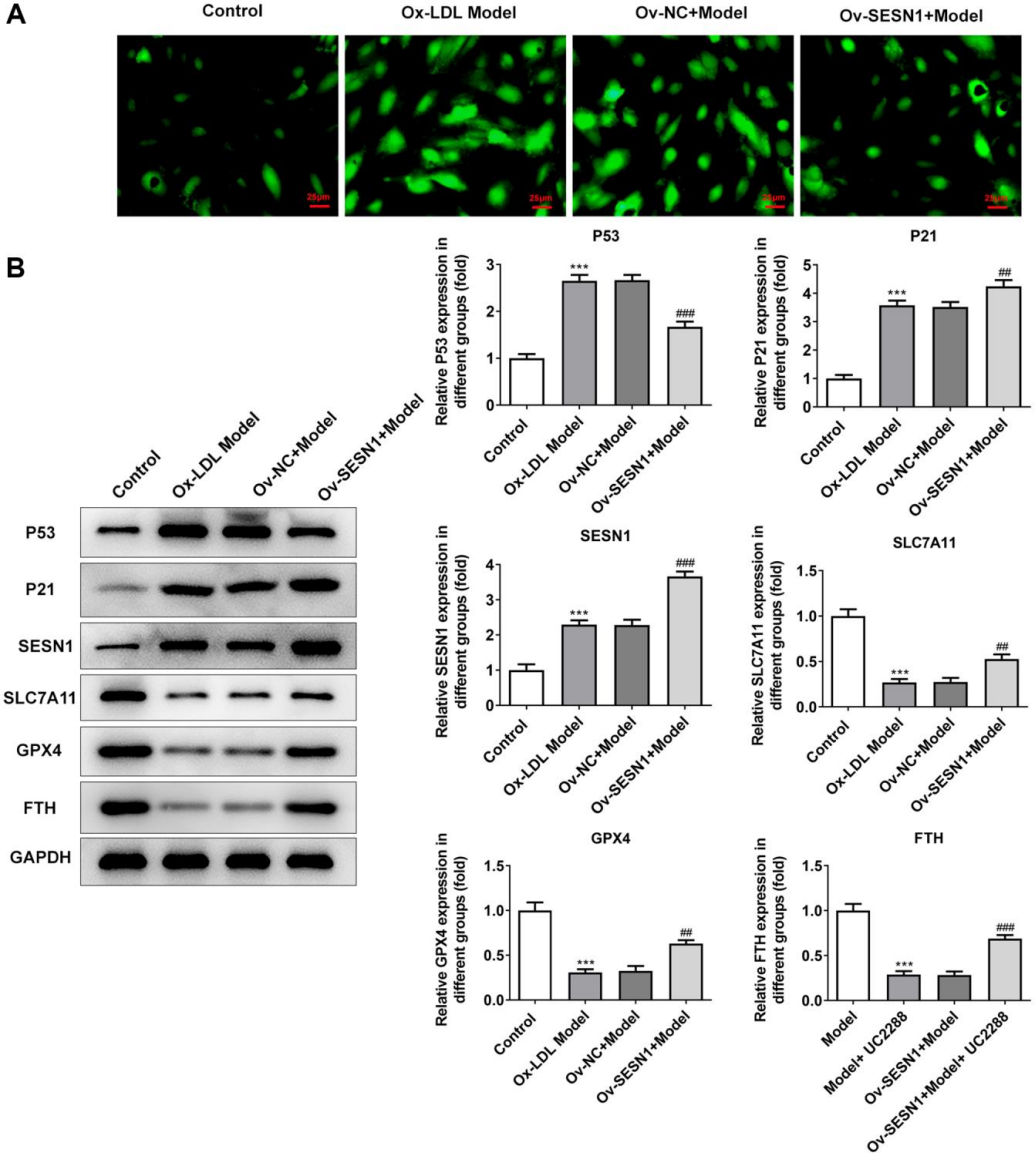
**Overexpression of SESN1 inhibited ferroptosis in ox-LDL-induced HUVECs.** (**A**) Immunofluorescence was used to detect the iron metabolism in vascular tissues. (**B**) The expressions of SESN1, P53, P21 and ferroptosis-related proteins were detected by western blot. ^***^P < 0.001 vs. Control; ^##^*P* < 0.01, ^###^*p* < 0.001 vs. Ov-NC+Model.

Immunofluorescence assay of total cell iron levels showed a significant increase following ox-LDL challenging and inhibition of total cell iron levels after SESN1 overexpression (Figure 5A). Western blot results showed that the expressions of SESN1, P53 and P21 were increased to varying degrees, while the expressions of SLC7A11, GPX4 and FTH were decreased significantly. Compared with the Ov-NC+Model group, the expression of P53 was decreased, the expressions of P21 and SESN1 were significantly increased, and the expressions of ferroptosis-related proteins were significantly increased in the Ov-SESN1+Model group (Figure 5B).

### Overexpression of SESN1 inhibited ferroptosis in ox-LDL-induced HUVECs via activating P21

**Figure 6:**
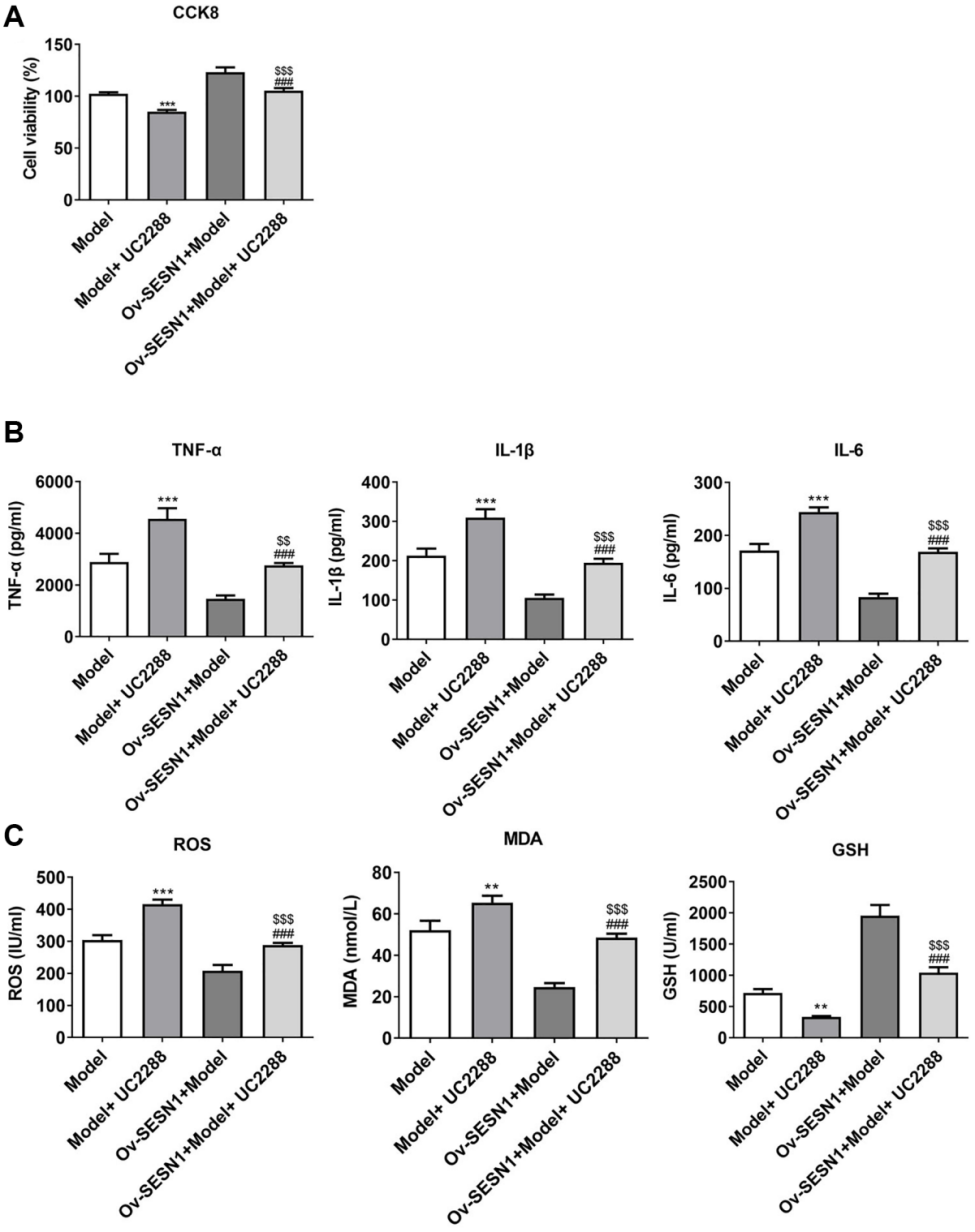
**Overexpression of SESN1 increased cell activity and inhibited cellular inflammation and oxidative stress responses in ox-LDL-induced HUVECs via activating P21.** (**A**) CCK8 was used to detect the cell activity. (**B**) ELISA was used to detect the levels of inflammatory factors in cells. (**C**) The kits were used to detect the expression of oxidative stress-related factors ROS, MDA and GSH. ^***^P < 0.001 vs. Model; ^###^*P* < 0.001 vs. Model+UC2288; ^$$^*P* < 0.01, ^$$$^*P* < 0.001 vs. Ov-SESN1+Model.

**Figure 7:**
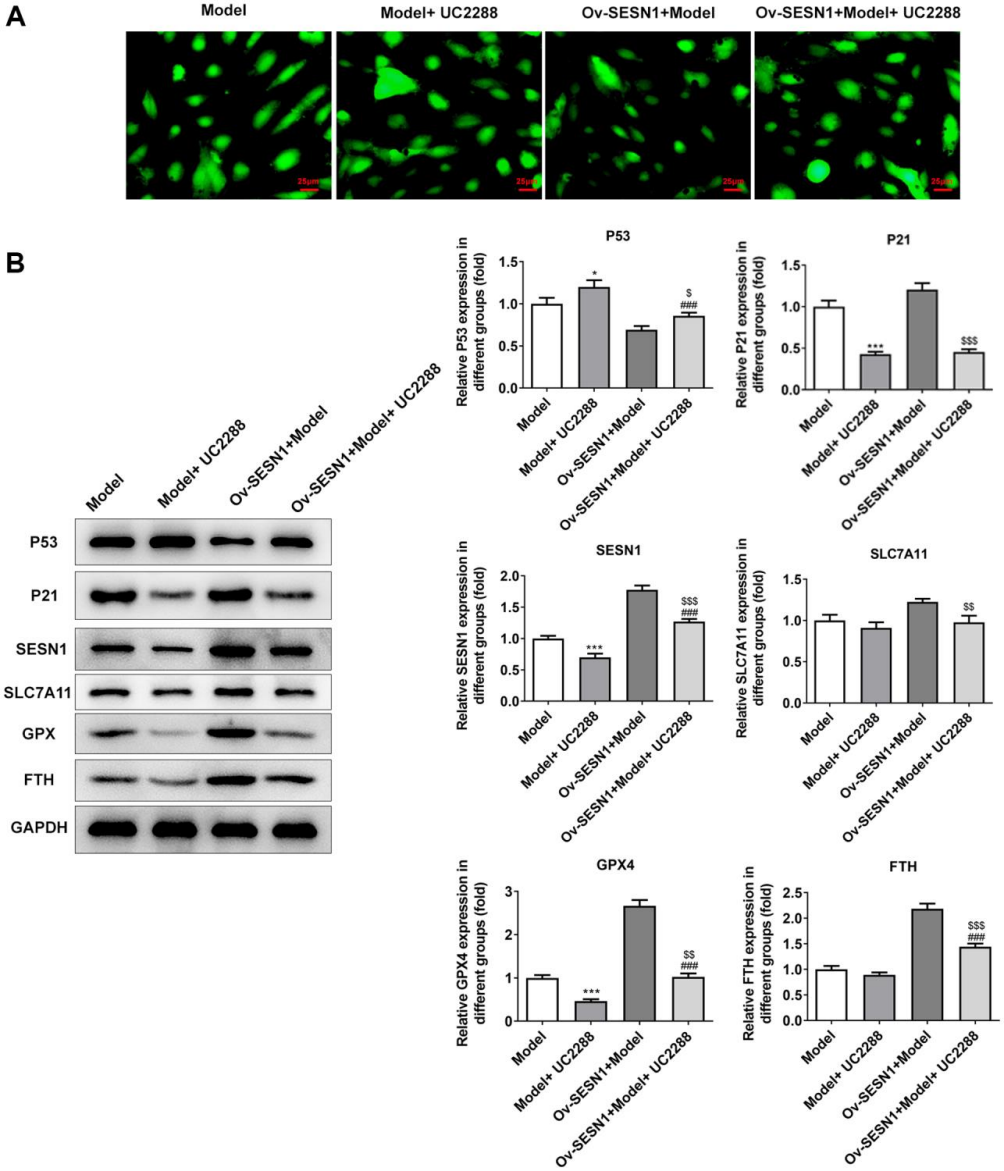
**Overexpression of SESN1 inhibited ferroptosis in ox-LDL-induced HUVECs via activating P21.** (**A**) Immunofluorescence was used to detect the iron metabolism in vascular tissues. (**B**) The expressions of SESN1, P53, P21 and ferroptosis-related proteins were detected by western blot. ^*^P < 0.05, ^***^P < 0.001 vs. Model; ^###^*P* < 0.001 vs. Model+UC2288; ^$^*P* < 0.05, ^$$^*P* < 0.01, ^$$$^*P* < 0.001 vs. Ov-SESN1+Model.

Next, the mechanism of SESN1 in regulating ferroptosis in AS was further explored. UC2288, a p53-independent inhibitor of P21, was added and the cells were divided into Model, Model+UC2288, Ov-SESN1+Model and Ov-SESN1+Model+ UC2288 groups. CCK8 results showed that the cell activity in model group was decreased significantly after UC2288 administration. Compared with Ov-SESN1+Model group, the cell activity in Ov-SESN1+Model+ UC2288 group was significantly decreased. However, the cell activity in Ov-SESN1+Model+UC2288 group was increased compared with that of Model+ UC2288 group (Figure 6A). ELISA results showed that compared with Ov-SESN1+Model group, the expressions of inflammatory factors in Ov-SESN1+Model+UC2288 group were increased. However, the expressions of inflammatory factors in Ov-SESN1+Model+UC2288 group were decreased compared with that in Model+UC2288 group (Figure 6B). The trend of ROS and MDA was consistent with that of inflammatory factors, while the trend of GSH was opposite to that of inflammatory factors (Figure 6C). Immunofluorescence analysis of total iron level showed that iron expression was increased in the Ov-SESN1+Model+ UC2288 group compared with the Ov-SESN1+Model group (Figure 7A). Western blot results showed that compared with the control group, the expression of P53 in the Model+UC2288 group was increased, while the expressions of P21 and SESN1 were decreased. Compared with the Model+UC2288 group, the expression of P53 in Ov-SESN1+Model+UC2288 group was decreased and the expression of SESN1 was increased. Compared with Ov-SESN1+Model group, the expression of P53 in Ov-SESN1+Model+UC2288 group was increased, and the expressions of P21 and SESN1 were decreased. Compared with the Model+UC2288 group, the expressions of GPX4 and FTH in Ov-SESN1+Model+UC2288 group were significantly increased. Compared with Ov-SESN1+Model group, the expressions of SLC7A11, GPX4 and FTH were significantly decreased in Ov-SESN1+Model+UC2288 group (Figure 7B).

## DISCUSSION

Sestrins are a family of highly conserved stress response proteins that are transcriptionally regulated by P53 and forkhead transcription factors and exhibit oxidoreductase activity to protect cells from oxidative stress *in vivo* [18]. SESN1 can inhibit ox-LDL-induced oxidative stress injury in endothelial cells, thereby inhibiting the development of AS. In addition, SESN1 is considered to be a downstream factor of aerobic exercise, which tends to inhibit the activation of inflammatory signals and thus the expression levels of inflammatory factors in AS [14]. Previous study has shown that SESN1 can inhibit oxidative stress damage of endothelial cells induced by ox-LDL, which might protect against AS [14]. Moreover, SESN1 has been shown to inhibit aortic inflammation and improve AS in a mouse model of AS [19, 20]. In our paper, we found that overexpression of SESN1 could significantly inhibit the formation of aortic plaque, inflammation and oxidative stress response, and thus improve the disease symptoms of AS.

SESN1 has been shown to be induced by hydrogen peroxide in a p53-dependent manner. However, the role of P53 in AS is diversified. On the one hand, P53 can inhibit the proliferation of smooth muscle cells and macrophages. In addition, mutant P53 is associated with the occurrence of AS, while wild-type P53 can reduce the inflammatory damage of AS [21]. On the other hand, in the AS model of ApoE−/− mice combined with high fat diet, P53 was significantly increased and correlated with high ferroptosis levels [22]. In addition, through the KEGG database, it is displayed that SESN1, which is activated by P53 transcription however, may play a negative role in the regulation of P53 through a series of feedback signal pathways. In our experiments, we found that the expression of P53 was abnormally increased in the animal models and cellular models of AS, and the expression of P53 was reversed after further overexpression of SESN1, which also confirmed that SESN1 was regulated by P53 transcription and negatively regulated P53 expression.

SESN1 is transcriptional regulated by P53 and has a physical binding effect on P21, activating P21 and its downstream signaling functions. P21 was predicted to be a downstream target gene of SESN1 by the STRING website (Supplementary Figure 1). In our paper, we found that the expression of P21 was significantly increased in the animal models and cellular models of AS. After overexpression of SESN1, the expression of P21 was further increased. Through literature review, P21 has been discovered to regulate the occurrence and development of ferroptosis in a p53-independent manner. This study also showed that overexpression of p21 reversed the iron sensitivity of cancer cells [23]. p21 can influence the aging of certain cancer cells by maintaining higher levels of ROS to regulate p53-ROS signaling [24]. These findings strongly suggest that P21 may play an important role in regulating ferroptosis. Moreover, targeted inhibition of p21 expression can promote endothelial cell proliferation, migration and vascular formation in peripheral arterial disease [25]. In this paper, we further explored the mechanism by adding the p53-independent inhibitor UC2288. Our results showed that the expression of P21 was inhibited by the addition of UC2288, while the decreased inflammatory response, oxidative stress response and ferroptosis due to SESN1 overexpression were reversed, further suggesting that SESN1 overexpression played a role in inhibiting vascular endothelial ferroptosis through the activation of P21.

## CONCLUSION

SESN1 overexpression plays an inhibitory role in vascular endothelial ferroptosis through the activation of P21 in AS.

## Supplementary Materials

Supplementary Figure 1
