# Supplementary material for: Regulation of endothelial ferroptosis by SESN1 in atherosclerosis and its related mechanism
Source: Aging (Albany NY). 2023 Jun 8;15(11):5052–65. doi: 10.18632/aging.204777 (PMC10292895; doi:10.18632/aging.204777)
Supplement: Supplementary Figure 1 [file aging-15-204777-s001.pdf]

SUPPLEMENTARY FIGURE

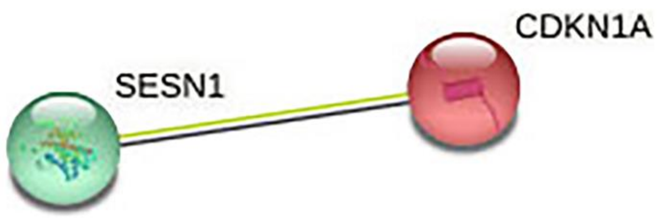

Supplementary Figure 1. P21 was predicted to be a downstream target gene of SESN1 by the STRING website.
